# Challenges of Carbapenem-Resistant *Enterobacteriaceae* in the Development of New β-Lactamase Inhibitors and Antibiotics

**DOI:** 10.3390/antibiotics14060587

**Published:** 2025-06-07

**Authors:** Pierre Leroux, Charleric Bornet, Jean-Michel Bolla, Anita Cohen

**Affiliations:** 1 Aix Marseille Université, INSERM, SSA, MCT, 13385 Marseille, France; pierre.leroux@etu.univ-amu.fr (P.L.); jean-michel.bolla@univ-amu.fr (J.-M.B.); 2 Pharmacy Department, Hôpital Conception, Assistance Publique-Hôpitaux de Marseille, AP-HM, F-13005 Marseille, France; charles-eric.bornet@ap-hm.fr; 3 Pharmaceutical Expertise and Clinical Research Unit, Pharmacy Department, Assistance Publique-Hôpitaux de Marseille, AP-HM, F-13009 Marseille, France

**Keywords:** *Enterobacteriaceae*, carbapenems, serine-β-lactamases, metallo-β-lactamases, diazabicyclooctanones, boronic acid derivatives

## Abstract

Nowadays, antimicrobial resistance (AMR) is a growing global health threat, with carbapenem-resistant *Enterobacteriaceae* (CRE) posing particular concern due to limited treatment options. In fact, CRE have been classified as a critical priority by the World Health Organization (WHO). Carbapenem resistance results from complex mechanisms, often combining the production of hydrolytic enzymes such as β-lactamases with reduced membrane permeability and efflux system induction. The Ambler classification is an effective tool for differentiating the characteristics of serine-β-lactamases (SβLs) and metallo-β-lactamases (MβLs), including ESβLs (different from carbapenemases), KPC, NDM, VIM, IMP, AmpC (different from carbapenemases), and OXA-48. Recently approved inhibitor drugs, such as diazabicyclooctanones and boronic acid derivatives, only partially address this problem, not least because of their ineffectiveness against MβLs. However, compared with taniborbactam, xeruborbactam is the first bicyclic boronate in clinical development with a pan-β-lactamase inhibition spectrum, including the IMP subfamily. Recent studies explore strategies such as chemical optimization of β-lactamase inhibitor scaffolds, novel β-lactam/β-lactamase inhibitor combinations, and siderophore–antibiotic conjugates to enhance bacterial uptake. A deeper understanding of the mechanistic properties of the active sites enables rational drug design principles to be established for inhibitors targeting both SβLs and MβLs. This review aims to provide a comprehensive overview of current therapeutic strategies and future perspectives for the development of carbapenemase inhibitor drug candidates.

## 1. Introduction

The issue of bacterial multi-drug resistance is no longer a hypothetical scenario but a concrete reality for healthcare systems. Since the discovery of penicillin by Alexander Fleming in 1928 [1], the marketing of an increasing number of antibiotics—up until the 1990s—has significantly contributed to the revolution in modern medicine [2]. Today, clinicians have access to a vast therapeutic arsenal of antimicrobials with varied spectra and pharmacological mechanisms to treat a wide range of bacterial infections [3]. However, the successive emergence of resistances to various pharmacological classes has led to, over the last few decades, the reality of Multi-Drug Resistant (MDR) and eXtensively Drug Resistant (XDR) species [4]. The fear of an uncontrolled increase in pan-drug-resistant strains has become a global public health issue. A 2021 study of 204 countries estimated that antimicrobial resistance (AMR) was responsible for 4.71 million deaths and directly attributable to 1.14 million deaths [5]. Projections are equally alarming, suggesting that by 2050, AMR-related mortality could reach 10.1 million deaths per year, surpassing the number of deaths currently attributed to cancer [5]. Beyond the human impact, studies also highlight the economic burden AMR could place on healthcare systems [6]. The increase in the number and length of hospital stays, as well as the growing use of costly therapeutic alternatives, could significantly worsen the current financial crisis [7]. Furthermore, the environmental consequences of excessive antibiotic consumption in human and veterinary medicine, as well as in intensive agriculture, have considerably upset the delicate balance of ecosystems [8]. The contamination of soil and water—both surface and ground—by antibiotic residues contributes to the selective pressure exerted on bacteria in the environment, creating numerous reservoirs of resistance genes [9]. This social, economic, and environmental triptych is at the heart of the One Health approach, which aims to tackle this problem through a systemic and global perspective [10]. This approach is more important given the rapid spread of resistance genes in a globalized world. Advances in bacterial genetics have revealed the presence of such genes in chromosomes and plasmid levels, whether constitutive or inducible [9]. The adaptability of bacterial genomes facilitates the emergence of new, resistance genes transmissible within the same bacterial ecosystem via various horizontal gene transfer mechanisms, including conjugation, transduction, and transformation [9]. As a result, the diversity of resistance mechanisms is considerable, involving enzymatic inactivation of antibiotics, overexpression of efflux pump, and modifications of biological targets [9]. Finally, the challenges and lack of investment by pharmaceutical companies further complicate the race against time to manage infections that were once routinely treatable [2,3,11,12,13,14]. The marketing of new antimicrobial agents is steadily declining, particularly against Gram-negative bacterial infections, which remain the most pressing therapeutic challenge [11,12,13,14,15,16].

## 2. Carbapenem-Resistant *Enterobacteriaceae* and the Diversity of β-Lactamases

*Enterobacteriaceae* are a family of Gram-negative bacilli belonging to the order *Enterobacterales*. Bacterial species resistant to antibiotics have been listed by the World Health Organization (WHO), classified as medium, high, or critical priority for the research and development of new antibiotics [13], and are currently grouped under the acronym ESKAPE-E: *Enterococcus faecium*, *Staphylococcus aureus*, *Klebsiella pneumoniae*, *Acinetobacter baumannii*, *Pseudomonas aeruginosa*, *Enterobacter* spp., and *Escherichia coli* [17,18]. Of these seven species, three belong to the *Enterobacteriaceae* family: *Klebsiella pneumoniae*, *Enterobacter* spp., and *Escherichia coli*. These pathogenic bacterial species are responsible for numerous infections, mainly in the digestive and urinary tracts. These physiological conditions are potential entry points for systemic complications such as bacteremia, sepsis, and even septic shock [19]. A number of studies have highlighted the impact of gut microbiota dysregulation, including iatrogenic causes [20,21], in the occurrence of CRE infections [22].

Because of their prevalence, *Enterobacterales* are a major target with a wide variety of resistance mechanisms [16]. *Enterobacteriaceae* resistant to third-generation cephalosporins and carbapenems, as an example, have been classified as critical priority by the WHO [13]. We will focus on the production of β-lactamases, in particular carbapenemases, by *Enterobacteriaceae*. The production of β-lactamases by other species, such as *Acinetobacter baumannii* [23] or *Pseudomonas aeruginosa* [24], will not be addressed in this review.

**Figure 1:**
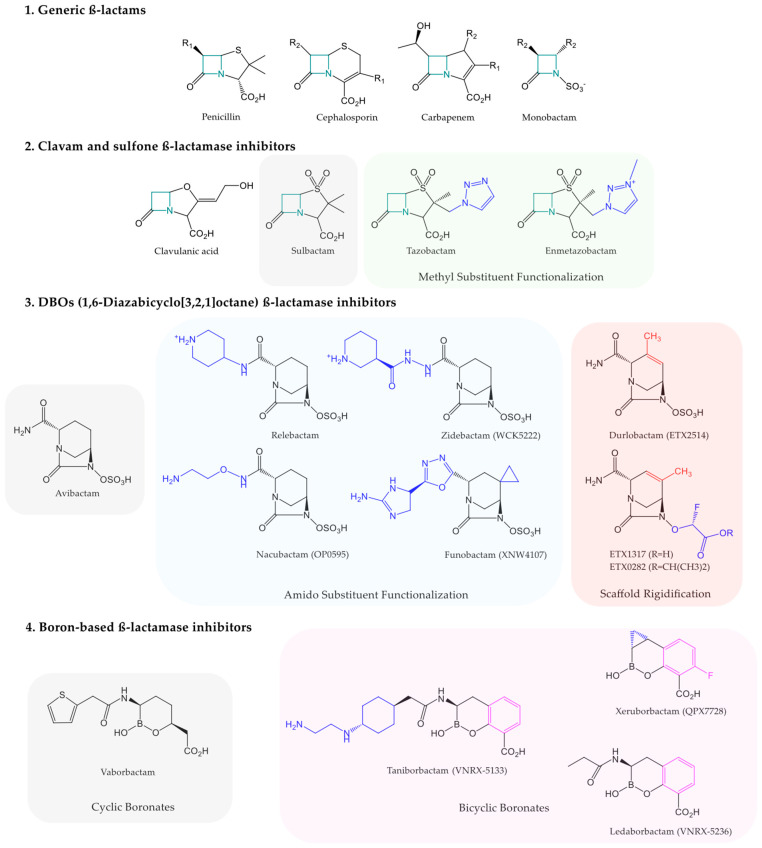
Representation of β-lactam scaffold (**1**) with the chemical structures of commercially available and clinically developing β-lactamase inhibitors, such as clavams and sulfone β-lactamase inhibitors (**2**), 1,6-diazabicyclo[3,2,1]octane β-lactamase inhibitors (**3**), and boron-based β-lactamase inhibitors (**4**). The lead compound of each class is highlighted in a gray area. Each pharmacomodulation is highlighted in colored areas.

β-lactamases are bacterial enzymes that hydrolyze the β-lactam ring, the pharmacophore of a class of commonly used antibiotics, the β-lactams [25]. Genes encoding β-lactamases can be found on chromosomes (non-horizontally transmissible) or plasmids (horizontally transmissible) [26]. β-lactams inhibit Penicillin-Binding Protein (PBP) transpeptidases, which are involved in peptidoglycan synthesis [27]. Peptidoglycan is a structural component of the cell walls of Gram-positive bacteria but is also found in the periplasmic space of Gram-negative bacteria. β-lactams are bactericidal antibiotics belonging to four families, each containing a four-membered azetidinone ring: penicillins, cephalosporins, carbapenems, and monobactams (Figure 1). They represent the treatment of choice for a wide range of bacterial infections, both as part of empirical therapy and as part of susceptibility-guided antibiotic therapy [27].

Within *Enterobacteriaceae*, there is a wide variety of β-lactam resistance mechanisms, including the acquisition of extended-spectrum β-lactamases (ESβLs) targeting penicillins, cephalosporins, and monobactams (with retained susceptibility to carbapenems and cephamycins), as well as inducible AmpC cephalosporinases [25,27]. It is also possible to isolate a group known as Carbapenem-Resistant *Enterobacteriaceae* (CRE). Meropenem, imipenem, and ertapenem are the carbapenems most widely used in clinical practice. Carbapenem resistance is polymorphic. It is often helpful to distinguish between Carbapenemase-Producing *Enterobacteriaceae* (CPE) and Non-Producing Carbapenemase Resistant *Enterobacteriaceae* (NP-CRE) [28]. NP-CREs are resistant to carbapenems by various, often complementary mechanisms. As an example, ESβLs expression is often associated with membrane impermeability. This reduced permeability of the outer membrane may result from alterations in porins and/or overexpression of efflux pumps [28]. However, we will mainly focus in this work on CPEs producing specific β-lactamases known as carbapenemases [25]. Five of these carbapenemases are of major medical interest: KPC, VIM, IMP, NDM, and OXA-48 [24,29,30,31]. In clinical practice, they are detected by CARBA NP, a colorimetric method marketed in the form of several rapid tests.

Ambler’s classification is a useful tool for classifying β-lactamases based on the amino acid sequence of their active site, providing an integrative understanding of the associated resistances [32].

Class A includes a wide variety of β-lactamases with a serine in their active site, also known as serine-β-lactamases (SβLs) [27]. Many enzymes are described, such as ESβLs, Guiana extended-spectrum β-lactamases (GESs), extended-spectrum β-lactamases active on cefotaxime (CTX-Ms), Sulfhydryl Variables (SHVs), and Temoneira (TEMs) [27]. Regarding carbapenemases, there is *Klebsiella pneumoniae* carbapenemase (KPC), which was historically discovered in a strain of *Klebsiella pneumoniae* in the United States. KPCs are now documented in a wide range of *Enterobacteriaceae* and have also been found in *Pseudomonas aeruginosa*. The prevalence of KPCs is high in the Americas, especially in the USA, Colombia, Argentina, and Brazil, although their spread in Europe has been increasing in recent years [33,34,35,36].

Class B includes metallo-β-lactamases (MβLs), which are carbapenemases containing at least one divalent zinc ion (Zn^2+^) in their active site [27]. Thus, MβLs can be classified based on the number of Zn^2+^ in their active pocket. Subclasses B1 and B3 contain two zinc atoms in their active pocket, while subclass B2 contains just one. The most common variants are Imipenemases (IMPs), Verona Integron-encoded MβL (VIM), and New Delhi MβL (NDM). The prevalence of MβLs is high in Asia, notably in India, China, Bangladesh, and Pakistan, as well as in Australia, the Middle East, and Europe [36]. As described below, this class is a major public health problem as none of the β-lactamase inhibitors currently on the market are effective against MβLs [33,34,35].

Class C primarily includes cephalosporinases with a serine in their active site, so they are also classified as SβLs. Notably, AmpC cephalosporinases are in this class [25,27].

Class D includes oxacillinases, which also have a serine in their active site [27]. This class includes some enzymes hydrolyzing carbapenems, such as OXA-48. OXA-48 is the most common carbapenemase in many European countries [36,37,38]. OXA-48-like enzymes such as OXA-12, OXA-163, OXA-181, and OXA-232 are also frequent in various *Enterobacteriaceae* species [33,34,35].

There are other classification systems for β-lactamases, such as the functional classification system of Bush–Jacoby–Medeiros [27].

## 3. Therapeutic Strategies, β-Lactamase Inhibitors, and New Antibiotics

Clinicians have a variety of therapeutic tools at their disposal to treat infections caused by ESβL-producing (extended-spectrum β-lactamase) bacteria. In the last few decades, many β-lactamase inhibitor drugs have been marketed. However, their spectrum of β-lactamase inhibition is heterogeneous.

β-lactamase inhibitor drugs can be classified according to their chemical structure (Figure 1). For instance, clavulanic acid, with a clavam structure, only inhibits certain class A penicillinases according to the Ambler classification. ESβL-producing pathogens are susceptible but they are not effective against class C AmpC cephalosporinases or carbapenemases of any class [27,39,40]. Similarly, penicillanic acid sulfone derivatives, such as sulbactam, tazobactam, and the recent enmetazobactam [27,38,40], share this limit [27]. These two chemical series of β-lactamase inhibitor drugs act as suicide substrates to SβLs, irreversibly inhibiting their target, particularly the serine in the active site of class A β-lactamases [27]. In contrast, most β-lactamase inhibitor drugs, as shown below, establish reversible covalent bonds with SβLs and may be regenerated, allowing multiple enzyme inhibitions per molecule [27].

The following β-lactamase inhibitor drugs are active against both ESβLs and AmpC. Regarding 1,6-diazabicyclo[3,2,1]octane analogs, avibactam has an inhibition spectrum covering KPC-class A carbapenemases and OXA-48-class D carbapenemases. Relebactam is only effective against KPCs and lacks activity against oxacillinases [16,27,28,39,40,41,42,43,44]. Similarly, the boronic acid derivative vaborbactam is only effective against KPCs [16,27,28,39,40,41,42,43,44]. Thus, no marketed carbapenemase inhibitor drug includes metallo-β-lactamases (MβLs) in its spectrum (Table 1).

New strategies combining these β-lactamase inhibitor drugs with a partner β-lactam are emerging [42]. As an example, the combination of ceftolozane/tazobactam (Zerbaxa^®^) is indicated to treat intra-abdominal infections and complicated pyelonephritis, as well as certain nosocomial pneumonia [27,45]. Tazobactam inhibits ESβLs and AmpC cephalosporinases but does not, in almost all the cases, restore ceftolozane activity against carbapenemase-producing strains. In contrast, the combination of ceftazidime/avibactam (Zavicefta^®^) is active against ESβLs, AmpC, KPC, and OXA-48 [27,39,45]. Such new combinations expand the spectrum of inhibition against critical pathogens such as MβL-producing *Enterobacteriaceae*. Strains producing MβL are known to remain sensitive to aztreonam, which is often hydrolyzed in the presence of other β-lactamases such as ESβLs or AmpC. The aztreonam/ceftazidime/avibactam or aztreonam/avibactam combination offers the advantage of treating complicated infections caused by MβL-producing *Enterobacteriaceae*, since avibactam protects aztreonam from coexisting ESβLs and AmpC. Research into new combinations is underway, to offer additional alternatives to imipenem/relebactam and meropenem/vaborbactam [27,45,46].

In addition to co-antibiotics, new antimicrobials have been developed against ESβL-producing bacteria (Table 1). This is the case with cefiderocol, which results from the covalent binding of a cephalosporin and a free iron-chelating siderophore. Cefiderocol is particularly effective in treating Gram-negative infections, working as a Trojan horse. The siderophore component chelates extracellular iron, allowing active, specific transport across bacterial outer membrane transporters [28,39,45,47]. Once in the periplasm, the cephalosporin component inhibits PBP transpeptidases, preventing peptidoglycan synthesis. Cefiderocol is thus effective against carbapenemase-producing strains (classes A and D) and some MβLs (such as VIM and IMP), though its activity is partial against NDM strains [41,46,48]. However, mutations in the target, transporters, bacterial iron metabolism, or β-lactamases have been documented as causes of cefiderocol’s loss of activity [49]. Additionally, due to its membrane-targeting mechanism, cefiderocol has little or no activity against Gram-positive bacteria [47].

Eravacycline, a newly marketed tetracycline, inhibits bacterial ribosomal subunit 30S, thereby impacting peptide synthesis. The lack of cross-resistance between eravacycline and carbapenems makes it a promising candidate for treating infections due to ESβL-producing bacteria, with relatively broad activity against carbapenemase-producing bacteria [38,39,41,45,46].

Plazomicin, an aminoglycoside approved by the FDA, also targets the bacterial ribosomal subunit 30S to inhibit protein synthesis. Its spectrum of inhibition against ESβLs is wide, and it is not affected by classical aminoglycoside-modifying enzymes (AMEs) such as acetyltransferases (AAC), phosphotransferases (APH), and nucleotidyltransferases (ANT). However, it may be ineffective against strains expressing 16S RNA methyltransferases, overexpressing efflux pumps like AcrAB-TolC, or underexpressing porins like OmpF. Plazomicin exhibits good activity against KPC and OXA-48-like serine carbapenemases, as well as partial inhibition against metallo-carbapenemases like IMP, VIM, and NDM [38,39,41,45,46,48].

Other membrane-targeting antibiotics, such as colistin, are also used to treat infections involving bacteria expressing ESβL, although their clinical use is affected by the nephrotoxicity and neurotoxicity associated with polymyxins [41]. Fosfomycin, commonly used orally for treating uncomplicated cystitis in women, is also being investigated for intravenous use, either alone or in combination, in the management of this type of infections [41,48,50]. Tigecycline, both alone and in combination, has demonstrated effectiveness against infections involving bacteria expressing ESβL, particularly in high-dose protocols, while avoiding the nephrotoxicity of polymyxins and aminoglycosides [38,39,41,46].

## 4. An Active and Multifaceted Clinical Research Landscape

Several β-lactamase inhibitor drug candidates are currently being investigated in various clinical trials. The development of new β-lactamase inhibitor drug candidates is primarily a result of the synthesis of analogs of already marketed inhibitor drugs [51,52]. This has recently been the case with the marketing of enmetazobactam, which belongs to the same family of penicillanic acid sulfone as tazobactam and sulbactam, widely used in clinical practice. The following new β-lactamase inhibitor drug candidates will be presented according to their inhibition spectrum within carbapenemases. However, it is important to note their efficacy against certain class A β-lactamases, such as ESβLs, and class C β-lactamases, such as derepressed AmpC.

The diazabicyclooctanone chemical family includes four β-lactamase inhibitor drug candidates in Phase III: nacubactam [27,40,45,46], zidebactam [27,38,40], durlobactam [27,40,45], and funobactam [40,53]. It is noteworthy that these molecules show promise in inhibiting SβLs. However, nacubactam exhibits only moderate activity against OXA-48-like enzymes. None of these inhibitor drug candidates exhibit activity against MβLs (Table 1).

Boronate derivatives include three β-lactamase inhibitor drug candidates: taniborbactam (Phase III) [27,38,40,45,46], xeruborbactam (Phase I) [40,45,54], and ledaborbactam (Phase I) [40]. These compounds show promise in inhibiting class A and class D SβLs. Both taniborbactam and xeruborbactam demonstrate inhibitory activity against MβLs NDM and VIM. However, only xeruborbactam displays partial activity against certain IMP enzymes, while taniborbactam does not. It is thus noteworthy that xeruborbactam is currently the only β-lactamase inhibitor drug candidate in development with activity against nearly all carbapenemases implicated in human pathology (Table 1).

To reduce hospital stays and the associated infectious risk with long-term intravenous therapy, the development of oral beta-lactamase inhibitors is also of major research interest. Currently in Phase I, ETX0282 is a diazabicyclooctanone pro-drug of ETX1317. This would be a promising drug candidate for the inhibition of class A, C, and D SβLs [45,55] (Table 1).

Several original antibiotics are also under clinical development, such as ancremonam (formerly LYS228), which is currently in Phase II. The rationale for developing this molecule is particularly innovative. Ancremonam is a derivative of aztreonam, a monobactam-class antibiotic. As previously described, aztreonam exhibits some activity against MβLs, which was an important feature to preserve during the development of ancremonam. The objective was to maintain inhibitory properties against NDM, VIM, and IMP while pharmacomodulating to ensure activity against SβLs. Ancremonam thus meets these objectives, also showing activity against KPC and OXA-48 enzymes [38,46] (Table 1).

New polymyxins are also under clinical investigation, such as SPR206 (Phase I), which would retain antimicrobial activity with reduced nephrotoxicity [45,47].

## 5. An Emerging and Promising Avenue of Exploratory Research

The importance of exploratory research is fundamental in the hope of increasing clinicians’ therapeutic arsenal in the next few decades. Research in drug discovery is particularly active in the fight against carbapenem-resistant *Enterobacteriaceae* (CRE).

One of the main strategies employed is the synthesis of β-lactamase inhibitor analogs [26]. The development of new carbapenemase inhibitors, as described above, is a perfect example. Regarding diazabicyclooctanone derivatives, the currently developed drug candidates mainly result from two pharmacomodulations of avibactam. On the one hand, various functionalizations of the amido substituent were carried out to develop the approved drug relebactam, as well as the drug candidates zidebactam and nacubactam, both in Phase III clinical trials. Durlobactam, on the other hand, is the result of a rigidification of the main scaffold [56]. As for boron-based inhibitors, the approved drug vaborbactam is a cyclic boronate. Boronic acid derivatives currently in clinical development, such as taniborbactam, xeruborbactam, and ledaborbactam, are bicyclic boronates that offer different conformation options to vaborbactam with the residues anchoring the active sites [56,57] (Figure 1). X-ray crystallography and in silico molecular modeling are currently used to study these mechanisms in detail.

Another strategy involves the development of original series of β-lactamase inhibitors resulting from screening campaigns and chemical hit-to-lead optimization. This is the case for ANT431, which aims to open the promising path of pyridine-2-carboxylic acid derivatives in the targeted inhibition of MβLs [27,58].

**Figure 2:**
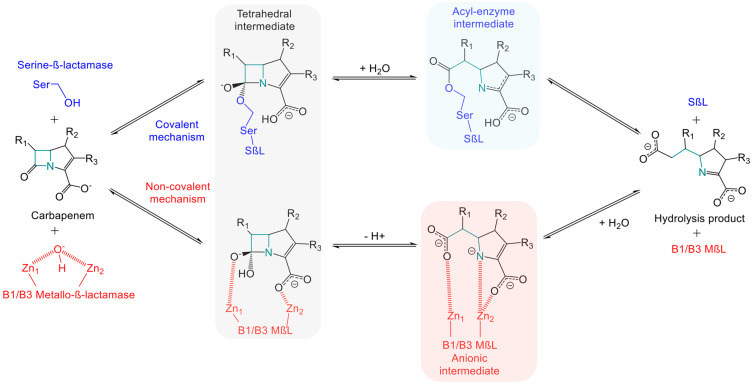
Schematic representation of hydrolytic mechanism of carbapenems by serine β-lactamases and B1/B3 metallo-β-lactamases. The β-lactam ring opening by B2 metallo-β-lactamases is not depicted here. This mechanism shares similarities with B1/B3 subfamilies, except for the presence of a single zinc Zn^2^⁺ ion in their active site.

Indeed, the therapeutic arsenal currently under development and available displays a weakness in fighting MβL-producing variants [59,60]. Furthermore, the documentation of strains producing both SβLs and MβLs highlights the urgent need to develop therapeutics effective against these strains too. To appreciate the difficulties involved in developing a pan-carbapenemase inhibitor, it is essential to understand the differential hydrolytic mechanism between these two types of enzymes [27]. Carbapenem hydrolysis by MβLs and SβLs is directly influenced by the amino acid sequence and three-dimensional architecture of their active site. Serine carbapenemases hydrolyze carbapenems via a nucleophilic substitution attack by the terminal hydroxyl group of serine residue on the carbon of the β-lactam ring carrying the carbonyl group. This results in the formation of a tetrahedral intermediate, leading to the opening of the β-lactam ring forming a covalent acyl-enzyme intermediate. The degradation product is released after cleavage of the ester bond covalently linking the serine residue to the carbon initially bearing the carbonyl group of the pharmacophore [26,27,61,62] (Figure 2). Hydrolysis by metallo-carbapenemases differs by using a non-covalent mechanism. The zinc ion is coordinated in the protein structure by three amino acids, which can vary depending on the enzyme and include cysteine, aspartic acid, or histidine. Subclasses B1/B3 use two zinc atoms to form a tetrahedral intermediate via the nucleophilic attack of a hydroxide ion or a water molecule on the carbon bearing the carbonyl function of the β-lactam ring. Conversion to a stable anionic intermediate occurs through coordination of the two zinc atoms with the carboxyl and carbonyl functions of the β-lactam ring, leading to its opening. The final degradation product is thus an inactive β-amino acid. Subclass B2 hydrolyzes β-lactams in a similar way to the subclasses B1/B3, except for the use of a single zinc atom. This zinc atom forms a non-covalent complex with the carboxyl group. However, a H_2_O molecule or OH^−^ anion interacts through weak energetic bonds with histidine, cysteine, and aspartic acid residues, leading to the degradation of the carbonyl group. The consequence is similar, with the opening of the β-lactam ring in the final inactive product [26,27,61,62] (Figure 2). The challenge in developing pan-β-lactamase inhibitors therefore lies in the ability to inhibit both enzymatic mechanisms. As mentioned above, some bicyclic boronates inhibit both MβLs and SβLs [59]. As an example, the boron atom in xeruborbactam enters into a covalent bond with the serine residue in KPC and OXA-48 for SβLs and with H_2_O molecules or OH^−^ anions for MβLs, such as NDM and VIM [54]. Other structures, besides boron-based inhibitors, have been described in the literature for multi-target inhibition. The acquisition of crystallographic structures of β-lactamases/substrates/inhibitors has led to advances in in silico techniques [57,63,64]. Modeling and molecular dynamics strategies now help to highlight the key elements of enzymatic mechanisms. Understanding these phenomena has led to the definition of specific criteria to guide the synthesis of inhibitors that meet the requirements of enzymatic hydrolysis by both SβLs and MβLs. Boron-based inhibitors therefore use a strategy of mimicking the tetrahedral intermediate described above [59,61]. Phosphonate-based inhibitors, azetidinimines, and azole-based inhibitors have been described using a dual non-covalent mode. This strategy mainly allows the phosphate or hydroxyl group of azetidinimines to establish hydrogen bonds with the hydroxyl groups of serine residues and electrostatic interactions with zinc-bearing anchor residues, replacing the H_2_O molecule. Azole-based inhibitors have also been described as using this mechanism via a tetrazole group [61]. Fusion of the pharmacophores led to mercaptoboronate derivatives using a covalent–non-covalent mode, with one part of the compound consisting of a boron atom forming a covalent bond with the hydroxyl group of serine in SβLs and another part containing a thiol group coordinating the two zinc atoms of MβLs [61]. Finally, the dual covalent mode of O-aryloxycarbonyl hydroxamates involves forming a double covalent bond with the hydroxyl group of serine residues and the amine function of lysine residues, which are well-documented in the active sites of MβLs [61].

The optimization of β-lactam drugs is also an ongoing research avenue. Increasing antimicrobial activity can be achieved by enhancing the fraction that hits the target. This can be realized through the classic strategy of testing original β-lactam/β-lactamase inhibitor combinations [42]. It can also resolve the problem of membrane impermeability and efflux induction often associated with β-lactamase synthesis in CRE. As described above, the monobactam chemical series, such as aztreonam, is a preferred molecule in the fight against MβLs. Thanks to the rational development of ancremonam (currently in Phase II), strategies have been presented to reduce hydrolysis of this antibiotic by class A β-lactamases. The innovative strategy proposed by cefiderocol to hijack bacterial iron metabolism and transport was also discussed. Some lines of research aim to conjugate siderophores with a known antibiotic pharmacophore, such as aztreonam [65].

Finally, in parallel with high-throughput screening campaigns aimed at identifying new targets, non-antibiotic treatment approaches have gained importance in the last few decades. This is particularly true of the development of monoclonal antibodies for infectious diseases. Bezlotoxumab is a perfect example of a monoclonal antibody in clinical use, targeting *Clostridium difficile* toxin B [66]. The challenge in developing monoclonal antibodies targeting enterobacteria lies in the fact that their pathogenicity is often non-toxin-mediated [47]. To improve access to the target, techniques involving the coupling of antibiotics with monoclonal antibodies are also being explored [47]. The discovery of natural bactericidal viruses has led to a resurgence of phage therapy, which could enable the selection of bacteriophages genetically modified to selectively induce lysis of CRE [46,67,68,69].

## 6. Conclusions

The fight against carbapenem-resistant *Enterobacteriaceae* (CRE) represents a major public health challenge. The globalization of human and material flows makes the dissemination of bacterial resistances such as serine-β-lactamases and metallo-β-lactamases easier worldwide. Their hydrolytic activity against carbapenems is particularly a cause for concern, raising fears of an increase in mortality associated with infections that were previously routinely treated. This also induces a significant financial burden on healthcare systems, with increased hospitalizations and extended stays, as well as the increased use of expensive therapeutics. The search for carbapenemase inhibitors is therefore crucial for the next few years. Recently approved inhibitor drugs such as diazabicyclooctanones and boronic acid derivatives only partially address this issue, notably due to their ineffectiveness against metallo-β-lactamases. However, while the pharmaceutical industry has been striving for decades to develop original antibiotic therapies specifically targeting Gram-negative bacteria, clinical and exploratory studies are more promising in the development of new β-lactamase inhibitor drug candidates. Among the molecules undergoing clinical trials, including the IMP subclass, xeruborbactam, compared to taniborbactam, is the first bicyclic boronate currently under clinical development with a broad-spectrum β-lactamase inhibition profile. Exploratory research also reveals innovative strategies optimizing these β-lactamase inhibitors by understanding the differences between the active sites of SβLs and MβLs. Research efforts are also focused on improving existing antibiotic structures. Cefiderocol, an integral part of the clinicians’ arsenal, and ancremonam, currently in phase II, are antibiotic therapies that perfectly illustrate the use of innovative mechanisms. Aztreonam, a monobactam, has excellent activity against metallo-β-lactamase-producing strains thanks to its structure. However, it is easily hydrolyzed by serine-β-lactamases such as ESβLs. Among the research directions, several strategies are under investigation to maintain aztreonam’s activity against metallo-β-lactamase-producing CRE strains while preventing its hydrolysis by serine-β-lactamases: new aztreonam/β-lactamase inhibitor combinations, chemical optimization, coupling with siderophores, etc. These techniques demonstrate the power of ongoing research in this area. In conclusion, challenges in drug development are increasingly marked by the need to combine advanced techniques in exploratory research. Today, the use of in silico screening based on the crystallographic structures of complexes is a valuable tool for future research.

## Figures and Tables

**Table 1:** Summary of antibiotic and β-lactamase inhibitor drugs and drug candidates, of interest for the main carbapenemases documented in CRE (carbapenem-resistant *Enterobacteriaceae*). The color code is as follows: green ✓ (total inhibitory activity), orange ≈ (partial inhibitory activity), and red ✕ (no inhibitory activity).

Carbapenemases		KPC	MβL (NDM, VIM, IMP)		OXA-48
Ambler classification		A	B		D
Active site		Serine	Zn^2+^		Serine
Geographical areas with high prevalence		America	Asia, Australia Middle East		Europe
β-lactamase inhibitor drugs					
Clavam	Clavulanic acid	✕	✕		✕
Penicillanic acid sulfone	Tazobactam	✕	✕		✕
	Sulbactam	✕	✕		✕
	Enmetazobactam	≈	✕		✕
Diazabicyclooctanone	Avibactam	✓	✕		✓
	Relebactam	✓	✕		✕
Boronic acid	Vaborbactam	✓	✕		✕
New antibiotic drugs					
Cephalosporin	Cefiderocol	✓	≈ (NDM)	✓ (VIM, IMP)	✓
Tetracycline	Evaracycline	✓	✓		✓
Aminoglycoside	Plazomicin	✓	≈ (NDM, VIM)	✓ (IMP)	✓
β-lactamase inhibitor drug candidates					
Diazabicyclooctanone	Nacubactam (Phase III)	✓	✕		≈
	Zidebactam (Phase III)	✓	✕		✓
	Durlobactam (Phase III)	✓	✕		✓
	Funobactam (Phase III)	✓	✕		✓
	ETX0282 (Phase I)	✓	✕		✓
Boronic acid	Taniborbactam (Phase III)	✓	✓ (NDM, VIM)	✕ (IMP)	✓
	Xeruborbactam (Phase I)	✓	✓ (NDM, VIM)	≈ (IMP)	✓
	Ledaborbactam (Phase I)	✓	✕		✓
Antibiotic drug candidates					
Ancremonam LYS228 (Phase II)		✓	✓		✓

## Data Availability

No new data were created or analyzed in this study.
